# Antibacterial properties and regenerative potential of Sr^2+^ and Ce^3+^ doped fluorapatites; a potential solution for peri-implantitis

**DOI:** 10.1038/s41598-019-50916-4

**Published:** 2019-10-09

**Authors:** A. D. Anastasiou, M. Nerantzaki, E. Gounari, M. S. Duggal, P. V. Giannoudis, A. Jha, D. Bikiaris

**Affiliations:** 1 School of Chemical and Process Engineering, University of Leeds, Leeds, LS2 9JT UK; 2 Sorbonne University, UPMC Univ Paris 06, CNRS, UMR 8234, PHENIX Laboratory, case 51, 4 place Jussieu, 75252 Paris cedex 05, France; 3 Laboratory of Polymer Chemistry and Technology, Chemistry Department, Aristotle University of Thessaloniki, 54124 Thessaloniki, Greece; 4 Laboratory of Biochemistry, Faculty of Medicine, Aristotle University of Thessaloniki, Thessaloniki, 541 24 Macedonia Greece; 5 Faculty of Dentistry, National University Centre for Oral Health, Singapore, Singapore; 6 Academic Department of Trauma and Orthopaedic Surgery, School of Medicine, University of Leeds, Leeds, LS2 9JT UK; 7 Department of Chemical Engineering and Analytical Science, University of Manchester, Manchester, UK

**Keywords:** Biomedical engineering, Biomaterials

## Abstract

Scaffolds and implants in orthopaedics and regenerative dentistry usually fail because of bacterial infections. A promising solution would be the development of biomaterials with both significant regenerative potential and enhanced antibacterial activity. Working towards this direction, fluorapatite was synthesised and doped with Sr^2+^ and Ce^3+^ ions in order to tailor its properties. After experiments with four common bacteria (i.e. *E*. *Coli*, *S*. *Aureus*, *B*. *Subtilis*, *B*. *Cereus*), it was found that the undoped and the Ce^3+^ doped fluorapatites present better antibacterial response than the Sr^2+^ doped material. The synthesised minerals were incorporated into chitosan scaffolds and tested with Dental Pulp Stem Cells (DPSCs) to check their regenerative potential. As was expected, the scaffolds containing Sr^2+^-doped fluorapatite, presented high osteoconductivity leading to the differentiation of the DPSCs into osteoblasts. Similar results were obtained for the Ce^3+^-doped material, since both the concentration of osteocalcin and the RUNX2 gene expression were considerably higher than that for the un-doped mineral. Overall, it was shown that doping with Ce^3+^ retains the good antibacterial profile of fluorapatite and enhances its regenerative potential, which makes it a promising option for dealing with conditions where healing of hard tissues is compromised by bacterial contamination.

## Introduction

Infections associated with bacterial colonisation around implants and scaffolds is a significant clinical problem both in orthopaedics and regenerative dentistry. Epidemiological studies suggest that between 12% and 43% of the dental implants will develop at some point symptoms of peri-implantitis when in orthopaedics, 2–5% of all the implant related procedures will be further complicated by bacterial infections^1^. Although so far, the use of antibiotics is a common practice for preventing or treating these conditions, the potential risk of antibiotic resistance is a growing concern and the effectiveness of their long-term use is disputable. In order to meet the critical clinical need against antibacterial resistance and overcome the long-term health implications of the current treatment strategies, there is an increased interest for the development of novel biomaterials with both intrinsic antimicrobial properties and the potential to trigger bone regeneration.

Calcium phosphates (CaPs), due to their structural and chemical similarity with the natural mineral of bone and dental hard tissues, are some of the most commonly used biomaterials for the fabrication of medical devices and implants^2–4^. Amongst them, hydroxyapatite [HAP: Ca_10_(PO_4_)_6_(OH)_2_], brushite (DCPD: CaHPO_4_·2H_2_O) and tricalcium phosphate [TCP: Ca_3_(PO_4_)_2_] are the most well documented, with also many reports concerning the use of fluorapatite (FAP: Ca_5_(PO_4_)_3_F) particularly in dentistry. Apart from the enhanced biocompatibility and bone regenerative potential, another advantage of such phosphate materials is the capability of tailoring their properties via anionic or/and cationic substitutions. Following that concept, Ca^2+^ ions can be partially replaced by metal ions like Ag^+^, Zn^2+^ and Sr^2+^ for imparting antibacterial properties to the biomaterials. However, the incorporation of these ions does not affect only the interaction of the materials with the bacteria but in many cases, it was found to have beneficial effects on the metabolism of tissue regeneration. Sr^2+^ and Ce^3+^ doped calcium phosphates are two characteristic examples of this behaviour.

Strontium can be found in dental enamel in concentrations less than 1000 ppb^5^. Sr^2+^ ions play a significant role during new bone formation by stimulating osteoblasts and simultaneous inhibition of bone resorption by suspending the function of osteoclasts. For this reason strontium modified calcium phosphate cements, have been explored for treating osteoporosis^6^, fixation of bone defects^7^, implant coatings and other biomedical applications^8–10^. Moreover, there are some reports to support that Sr containing minerals present antibacterial activity. Guida, *et al*.^11^, investigated the effect of Sr^2+^ in glass ionomer cements and observed bacterial inhibition with the release of Sr^2+^ ions. Strontium containing hydroxyapatite was also found to have particularly improved antibacterial effects on *Escherichia coli*, *Staphylococcus aureus* and *Lactobacillus*, showing potential to anti-caries applications in dentistry^12,13^. However, based on other reports the antibacterial potential of Sr^2+^ ions, is disputable. For example, Kumar *et al*.^14^, demonstrated that Sr^2+^-doped calcium phosphate nanoparticles show a weak antibacterial effect on *Staphylococcus aureus* and no effect on *Escherichia coli* even at high concentrations (i.e. 300 μg/μl) in contrast with Ag^+^ doped nanoparticles which proved to have clear antibacterial impact.

Cerium is a rare earth element which also has been used in biomaterials to stimulate antibacterial activity. In a recent work, Gopi *et al*.^15^, investigated the synergistic actions of Sr^2+^ and Ce^3+^ in hydroxyapatite nanoparticles. The results indicated that Ce^3+^ doped particles show extremely strong effect on *Escherichia coli* and *Staphylococcus aureus* even at low concentrations. Furthermore, in other works cerium was investigated to have significant pharmacological potential due to the antioxidant properties of Ce^3+^ and Ce^4+^ ions^16^ and although the exact role of cerium (Ce-ions) to the metabolism is still unclear, there is evidence that link it with angiogenesis through regulation of the intracellular oxygen^17^.

In the present work Sr^2+^ and Ce^3+^ doped fluorapatites (FAP) were synthesised in order to evaluate the antibacterial properties and regenerative potential, targeting to provide improved biomaterials for dental related applications. FAP was chosen over other calcium phosphates, based on the beneficial effect of fluoride on enamel. Absorption of the fluoride ions from enamel crystals is considered to offer protection from demineralisation^18^ and at the same time it appears to have some potential to prevent the attachment of bacteria due to the presence of F^−^ ions. Moreover, FAP is more stable than natural enamel in acidic conditions as demineralisation of fluorapatite begins at pH lower than 4.7, in contrast with the hydroxyapatite in enamel, which is more acid soluble (below pH = 5.5) due to the substitution of carbonate ions in the mineral crystal lattice^2^. The antibacterial properties of the synthesized materials in powder form, were evaluated after experiments with four common bacteria i.e. *Escherichia coli*, *Staphylococcus aureus*, *Bacillus subtilis* and *Bacillus cereus*. To test the regenerative potential, the mineral powders were used as fillers in chitosan scaffolds. Chitosan is a natural polymer and has been widely used as the main scaffolding material both for bone regeneration^19^, and for restorative dental related applications like the restoration of periodontal tissues^20^. During our experiments, dental pulp stem cells (DPSCs) have been cultured on the mineral containing scaffolds for one month. Different tests have been conducted to investigate the biocompatibility of the materials and the potential of DPSCs to differentiate into osteoblasts.

## Materials and Methods

### Synthesis of fluorapatites and chitosan scaffolds

The synthesis of the fluorapatite minerals followed the precipitation method described in^21^ and^22^. In particular, 200 ml of 0.1 M Ca(NO_3_)_2_∙4H_2_O aqueous solution, was prepared (solution A) and then heated to 50 °C. After stabilizing the temperature, 200 ml of a 1 M (NH_4_)_3_PO_4_ solution (solution B) containing the appropriate mass of NH_4_F, based on the stoichiometry of fluorapatite, was introduced dropwise to solution A. To maintain pH above 9, 0.1 M molar strength solution of NaOH was added dropwise during the mixing of A and B. The final mixture was left for 2 hours by continuous stirring at 50 °C for 2 h and then left to settle for 1 h to allow precipitation of fluorapatite. During precipitation, the top of the beaker was sealed with aluminum foil for minimising ingress of CO_2_ into the phosphate mineral solution. The formed fluorapatite crystals, were collected on a filter paper (Whatman grade 44 with pores of 3 μm), washed several times with distilled water and then dried for 24 h at 80 °C. After filtration the powder was heat treated to 400 °C for 3 h. For the synthesis of the Sr^2+^ and the Ce^3+^ doped FAP, 5% mol of Sr(NO_3_)_2_ and 5%mol of Ce(NO_3_)_3_·6H_2_O respectively, were added into solution A before the dropwise addition of the (NH_4_)_3_PO_4_. Stirring, filtration and drying followed as it is described for the synthesis of the un-doped material. All the minerals used in the present study are presented in Tables 1 and 2.

**Table 1:** Synthesized materials and expected chemical formulas.

Code name	Sr % mol.	Ce % mol.	Chemical formula
FAP	—	—	Ca_10_(PO_4_)_6_F_2_
FAP-Sr	5	—	Ca_9.5_Sr_0.5_(PO_4_)_6_F_2_
FAP-Ce	—	5	Ca_4.75_Ce_0.25_(PO_4_)_3_F_1.5_O_0.5_

**Table 2:** Fabricated chitosan scaffolds.

Code name	Chitosan to acetic acid ratio	Filler mineral (ratio based on the chitosan)
CH	*3*% *w*/*v* (*same concentration for all scaffolds*)	No fillers
CHFAP		20% w/w FAP
CHFAP-Sr		20% w/w FAP-Sr
CHFAP-Ce		20% w/w FAP-Ce

For the fabrication of the chitosan-fluorapatite scaffolds, the fluorapatite powder (20% wt in respect with chitosan) was dispersed in 2% v/v aqueous acetic acid solution. The mixture was processed in a sonicating bath for 10 min for homogenous dispersion of the particles. High molecular chitosan powder was added into the solution (3%wt/v) and left to dissolve under continuous stirring at 50 °C. The chitosan/mineral mixture was injected into the 1 cm diameter cell cultivation wells and placed in a freezer overnight (−20 °C). The frozen samples were transferred into a freeze-dryer where they were held for 24 h until each sample was dry. To remove any traces of the solvent, the scaffolds were immersed in 1 M NaOH solution for 5 min and then washed with a phosphate buffer at pH 7.4. Treating chitosan scaffolds with NaOH, deprotonates and forms new hydrogen bonds, which helps in stabilising the chitosan scaffolds in aqueous environment by lowering their dissolution rate.

### Characterisation techniques

A Bruker D8 powder diffraction spectrometer, with monochromatic Cu Kα radiation (0.1541 nm) was used for ascertaining the crystalline nature of the synthesized fluorapatite mineral powder. The diffractometer step size was 0.065° and the 2θ scanning range was from 10° to 60°, such that data were collected over a period of approximately 25 min with a scan speed of 0.014° s^−1^.

Scanning electron microscopy (SEM, a Hitachi SU8230 1–30 kV cold field emission gun) was used to investigate the size and shape of the synthesized crystals and also for characterising the porosity in chitosan scaffolds. The microscope was equipped with an Oxford Instruments 80 mm^2^ SD detector for energy dispersive X-ray (EDX) spectrometry with Aztec processing software to enable compositional analysis both of the mineral powders and the scaffolds. Since the calcium phosphate minerals have poor electrical conductivity, prior to SEM and EDX analysis, it was necessary to coat each sample with a 5 nm thick layer of iridium and then vacuum clean them for 10 min.

The FTIR spectra of the fluorapatite minerals and the chitosan scaffolds were acquired using an ATR-FTIR spectrometer (Bruker Vertex 70) for a range of wave numbers between 400 and 3000 cm^−1^.

### Antibacterial properties and cell cultivation experiments

The antibacterial activity of the mineral powders was evaluated according to Varna, *et al*.^23^. Bacterial strains of *E*. *coli* (BL21) were used as the model Gram-negative bacteria and strains of *S*. *aureus* (ATCC 25923), *B*. *cereus* and *B*. *subtilis* were used as Gram-positive bacteria. The microorganisms left to grow in 100 ml of sterile nutrient broth (Luria-Bertani broth, LB) at 150 rpm and 37 °C. They were collected at the logarithmic stage of growth and formed suspensions with the addition of 25 mM phosphate buffered saline (PBS, Sigma). The concentration of each suspension was adjusted to OD600 (optical density at 600 nm) value of 0.5 before incubation with the respective powder sample. Each mineral was tested for three different concentrations namely 25, 50 and 100 μg/ml. After 24 h the absorbance was measured at OD600 in triplicates and in each case, we obtained the mean value for the reported data (average values ± SD). The IC_50_ values for each material-microorganism pair have been calculated based on the linear regression model.

### Dental pulp stem cells (DPSC) isolation and culture

All the cell experiments were performed in accordance with relevant guidelines and regulations. Aiming to the isolation of DPSCs, upon extraction, the human deciduous tooth was received in 0.12% chlorexidin contained in Hank’s balanced solution and double washed with 2% penicillin-streptomycin (Pen/Strep) (Biowest) solution. By restraining the tooth with the help of a pincher and the use of multi-dimensional neuroextractors, the dental pulp was removed and placed in a solution of 4 mg/ml collagenase and 2 mg/ml dispase (SIGMA) dissolved in PBS and subsequently stirred for 45 minutes in a moving incubator in 37 °C. A complete α-MEM media supplemented with 100 mg/ml Pen/Strep, 2 mM L-glutamine, 0.1 mM L-ascorbic acid and 15% Fetal Calf Serum was added in a final volume of 10 ml resulting in centrifugation at 700 rcf, for 10 minutes in 20 °C. The pellet was resuspended in 2 ml of the same media and was finally plated in one well of a 6-well plate starting the cell culture in an incubator in 37 °C with 5% CO_2_ supply and media changes every 2–3 days upon confluence.

### Preparation of scaffolds and cell plating

The fabricated scaffolds were sterilized in gradually reduced ethanol concentration solutions and after washing three times with distilled H_2_O were left to dry for approximately 4 hours under sterile conditions.

Fibrin glue was prepared following the blood sampling of a healthy volunteer donor as described previously in^24^ and used for covering the bottom of 48 well plates (15 μl per well). The materials were placed in the wells using a sterile pincher by applying minimal manual pressure and left to dry until complete adherence upon the plastic surfaces. DPSCs were detached via 0.05% Trypsin-EDTA (Biowest) and counted with Trypan Blue in a Neubauer cell counting chamber. 3 ∙ 10^5^ cells were resuspended in α-MEM media supplemented with 10% FBS, penicillin (100 IU/ml) and streptomycin (100 lg/ml) and were subsequently injected per scaffold using an insulin syringe with an 18 G needle. Upon a 2-hour incubation, 500 μl α-MEM full medium were added per well for the initiation of culture upon the scaffolds.

### Cell viability assays (MTT assay and preparation for SEM)

Aiming the evaluation of the scaffold cytotoxicity, an MTT assay was performed (Sigma-Aldrich) 48 h after the initial plating of the DPSCs on the scaffolds. As a control group, non-plated DPSCs, were used in the same number as the rest of the groups. Briefly, after removing the media from the wells, MTT reactant was added in 1:10 ratio with DMEM media followed by a 4 h incubation in 37 °C and 5% CO_2_. The MTT was removed and 1 ml/well of DMSO was introduced for another one hour of incubation in the same conditions. The reduction of MTT to formazan was estimated at 570 and 630 nm wavelength.

The observation of DPSCs morphology and differentiation capacity after 6 days upon co-culture on the scaffolds was performed after their fixation with 4% Paraformaldehyde for 20 min in RT and overnight air dry.

### Quantification of secreted osteocalcin

The levels of the secreted osteocalcin in the culture supernatants was performed using a Human Osteocalcin Elisa kit (SIGMA) 15 days after the initial plating and according to the manufacturer’s instructions. The quantification was materialized in a Perkin Elmer VICTOR™ Multilabel Plate Reader and expressed in pg/ml. As a control group, the supernatant of a simple DPSCs culture, non-plated upon scaffolds, was used.

### Real time PCR (RUNX2 expression)

In order to compare the expression levels of genes correlated with differentiation to osteoblasts (RUNX2) in multi-treated scaffolds, real-time PCR was performed using a KAPA SYBR® FAST one step qPCR Master Mix (2X) Kit. In detail, 15 days after the DPSCs culture upon scaffolds, the latter ones were washed in PBS solution and the incorporated cells were detached using 0.05% Trypsin-EDTA (Biowest) and subsequent washes. RNA extraction and RNA quantification in a NanoDrop ND-1000 UV-Vis Spectrophotometer was carried out according to the manufacturer’s instructions. Upon the cautious primer designing (Table 3) and HPLC purification, for 10 ng of RNA template, Q-PCR was performed, the results of which were analysed using a ddCt algorithm in order to calculate the relative changes in gene expression.

**Table 3:** Primers designing for qPCR.

Gene	Forward Primer	Reverse primer
β-actin	atctggcaccacaccttctacaatgagctgcg	cgtcatactcctgcttgctgatccacatctgc
RUNX2	ttacttacaccccgccagt c	tatggagtgctgctggtctg

### Ethical statement

This study was approved by the Ethics Committee of Aristotle University of Thessaloniki School of Medicine (390-9/1.7.2017). This research involves Human Participants after their informed consent. Human deciduous teeth for mesenchymal stem cells isolation were collected from healthy volunteer donors during stem cell banking in Biohellenika SA Biotechnology Company. All the experiments regarding the cell work were performed in accordance with relevant guidelines and regulations.

## Results

**Figure 1:**
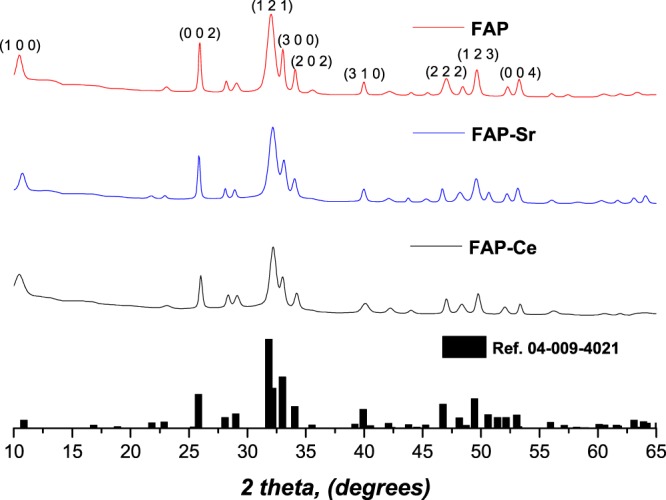
X-ray diffraction patterns of the fluorapatite minerals and comparison with a reference pattern for fluorapatite.

In Fig. 1 the X-ray powder diffraction data for the synthesised mineral powders are compared with the reference pattern of hexagonal fluorapatite (Ref. 04-009-4021). It is obvious that all the major peaks (for 2θ = 10.46, 25.95, 32.04, 33.02, 34.07, 39.98, 47.06, 49.67 and 53.26) match completely with the reference and consequently, these three materials are confirmed to be fluorapatite minerals. The incorporation of Sr^2+^ and Ce^3+^ into the fluorapatite lattice does not seem to affect the crystallographic identity of the materials since both the un-doped material (FAP) and the doped ones (FAP-Sr and FAP-Ce) found to have very similar (but not identical) XRD patterns (same position and relatively intensity of the peaks). The most significant difference is the peak that appears at 2θ = 50.5 for the FAP-Sr. Although this is not present neither to FAP or FAP-Ce it still can be found to the reference pattern of fluorapatite (Fig. 1). For the FAP-Sr was also observed a shifting of the peaks to lower angles by 0.2 degrees. The lack of significant alterations in the lattice dimensions of fluorapatite may be explained by considering the ionic radius of Sr^2+^ (132 pm) and Ce^3+^ (114 pm), which are comparable with that of Ca^2+^ (114 pm), suggesting that the effect of cationic substitution on lattice parameter is insignificant.

**Figure 2:**
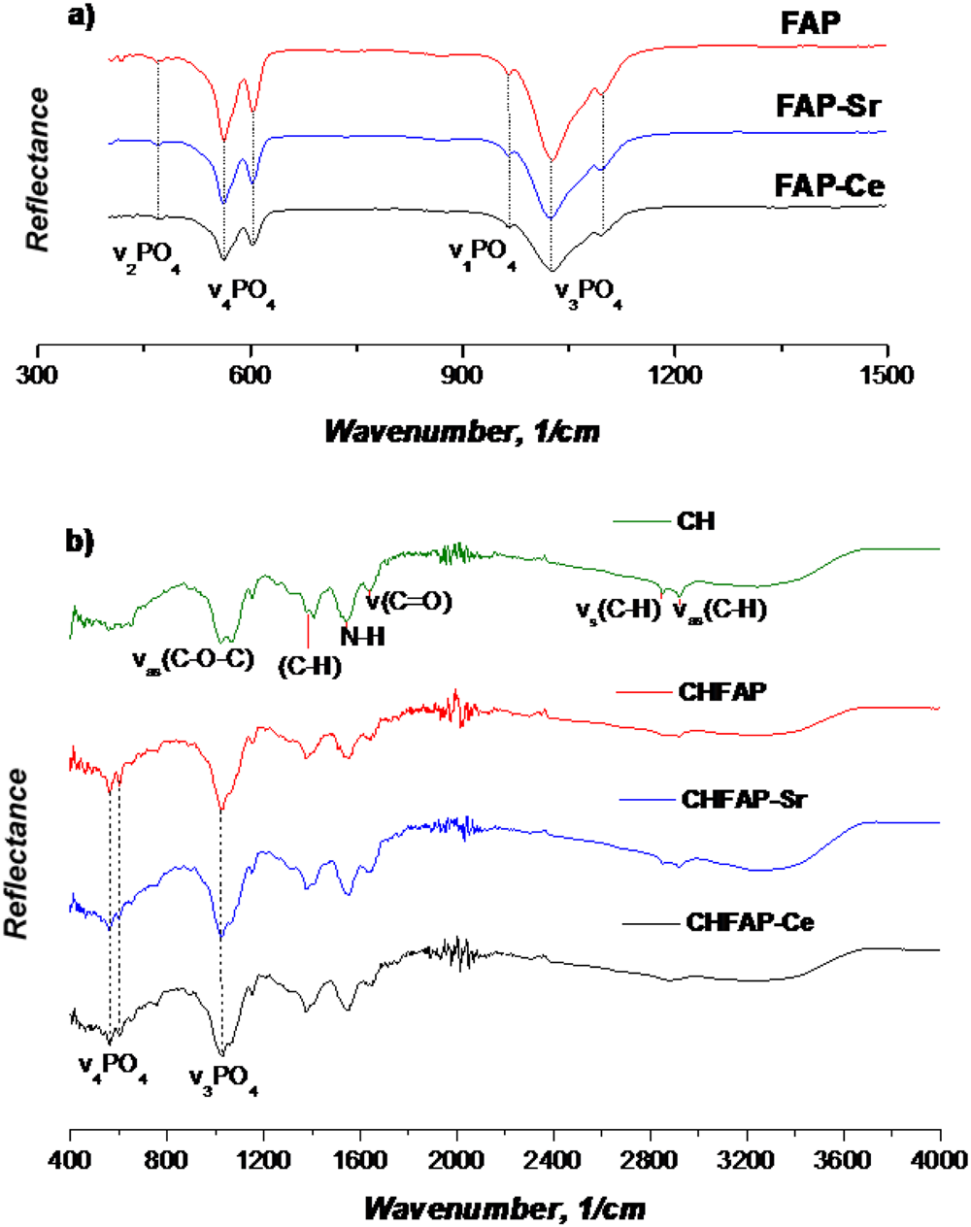
FTIR patterns of the fabricated materials; (**a**) patterns for the fluorapatite minerals (wavenumber range 300–1500 cm^−1^; (**b**) patterns of the chitosan scaffolds with and without minerals (wavenumber range 400–4000 cm^−1^).

The FTIR spectra of the mineral powders and chitosan scaffolds are compared in Fig. 2a,b, respectively. All the fluorapatites present similar peaks attributed to the PO_4_^3−^ ion bands. Specifically, the v_2_ band was identified at 468 cm^−1^, v_4_ at 559 and 600 cm^−1^, and the v_1_ band at 962 cm^−1^. The higheste peak was found at 1026 cm^−1^ and is assigned to the v_3_PO_4_ ion band (Fig. 2a). For the chitosan scaffolds, we observed peaks at 2918 cm^−1^ and 2854 cm^−1^ due to the C-H stretch, 1637 cm^−1^ for the C=O streching, 1535 cm^−1^ (N-H stretch), a broad peak between 1409 and 1369 cm^−1^ due to C-H bending and a double peak at 1031 and 1074 cm^−1^ due to C-O-C bridge^25^. The presence of minerals in CHFAP, CHFAP-Sr and CHFAP-Ce is verified with the peaks at 559 cm^−1^, 600 cm^−1^ and 962 cm^−1^ which are assigned to the PO_4_^3−^ ion bands as discussed before (Fig. 2b). The incorporation of mineral phase in chitosan changes the shape of peaks in the 800 to 1200 cm^−1^ range, suggesting that there might be molecular level interaction between the C-O-C and PO_4_^3−^ groups.

**Figure 3:**
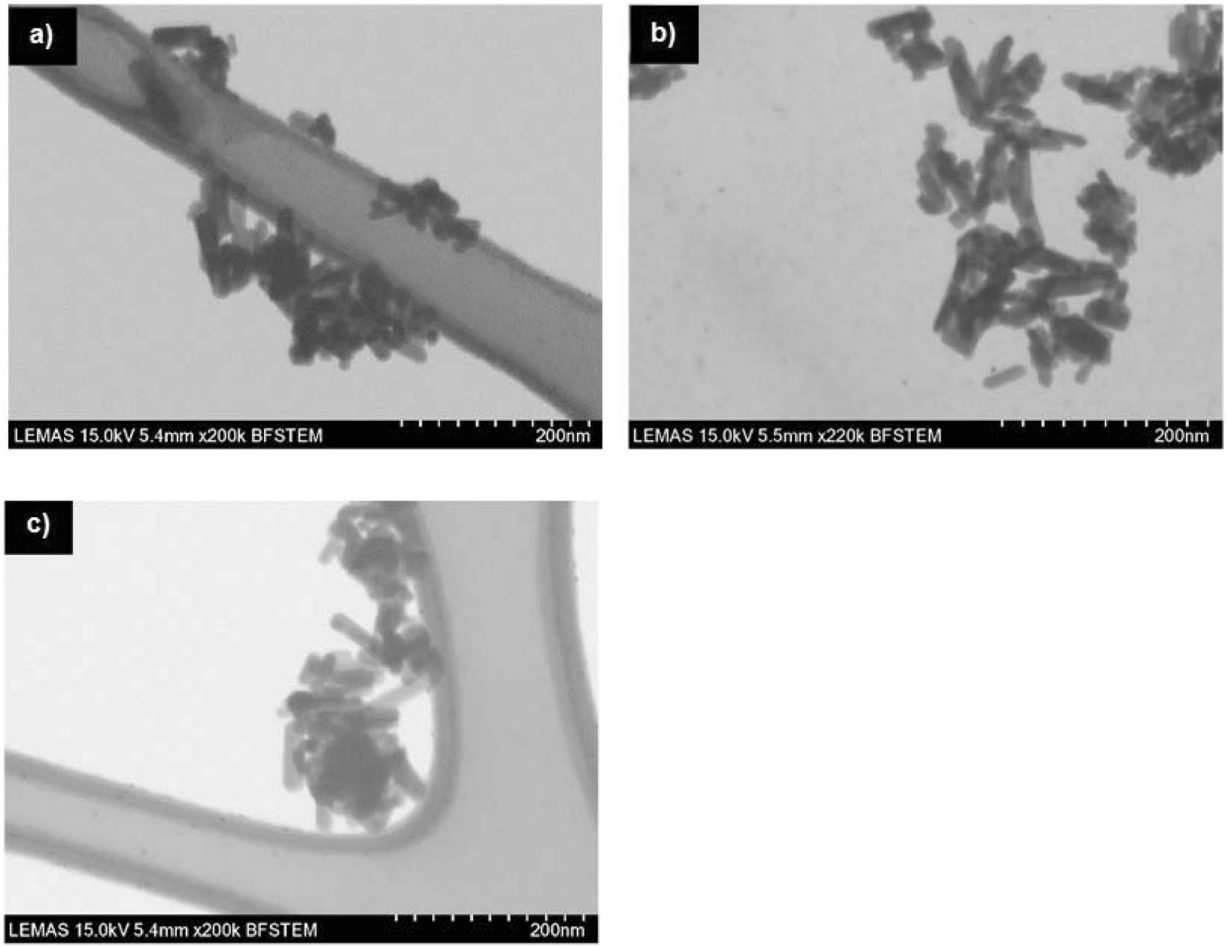
SEM images of the synthesised fluorapatite minerals (powder form); (**a**) un-doped fluorapatite (FAP); (**b**) 5% Sr doped fluorapatite (FAP-Sr); (**c**) 5% Ce doped fluorapatite (FAP-Ce).

The shape morphologies of fluorapatite crystals are presented in Fig. 3a–c where its verified that there aren’t any differences between the un-doped and the doped materials since all three minerals apear to constitute from rod like crystals, which are about 60 nm long (Fig. 3a,c). During STEM, in all samples only the fluorapatite crystals were identified which means that doping with Sr^2+^ and Ce^3+^ is not causing the formation of any secondary phases (e.g. metal nanoparticles) and consequently, it is assumed that these ions are incoprorated into the fluorapatite lattice.

**Figure 4:**
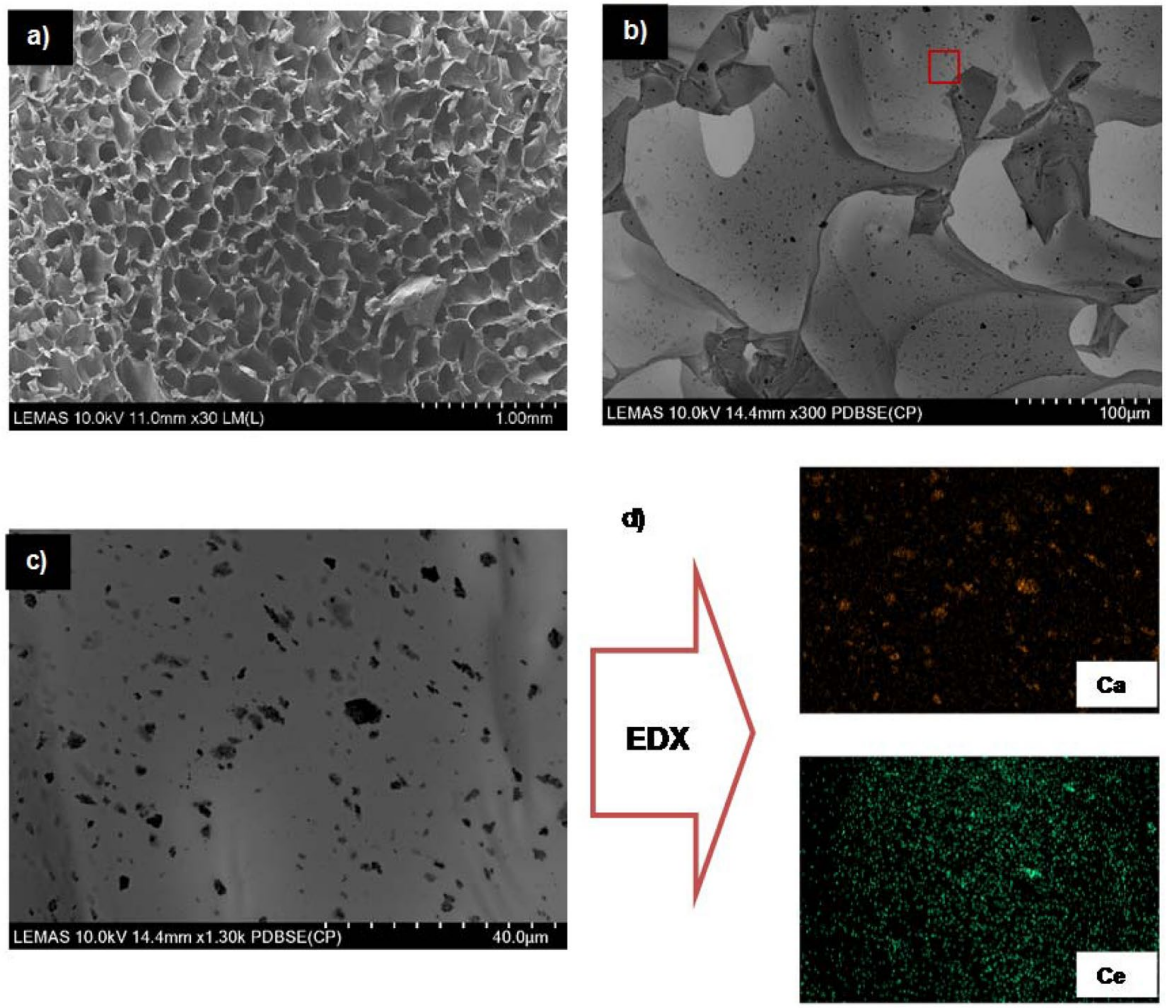
Typical SEM images of the chitosan scaffolds (images from the **CHFAP-Ce**). (**a**) Porosity at the surface of the scaffold; (**b**) distribution of the mineral nanoparticles in the scaffold; (**c**) mineral nanoparticles at higher magnification; (**d**) typical EDX maps of Ca and Ce.

In Fig. 4 typical SEM images of a mineral containing scaffold are presented, which show an extended network of pores with size range between 100 and 250 μm in diameter. These pores have been evolved after freeze drying of the chitosan solution (Fig. 4a). Although the role of porosity in bone regeneration is well known, the optimal pore size is not yet defined and as a general rule of thumb, porosity within a range between 50 and 400 μm is reccomended^26^ and thus the pore size and distribution shown in Fig. 4a is consistent with the requirements for cell growth. As it is depicted in Fig. 4b,c, aggregates of crystalline minerals with an average diameter <1 μm are homogenously distributed in a chitosan matrix, which can also be verified by EDX mapping (e.g. Ce in Fig. 4d).

**Figure 5:**
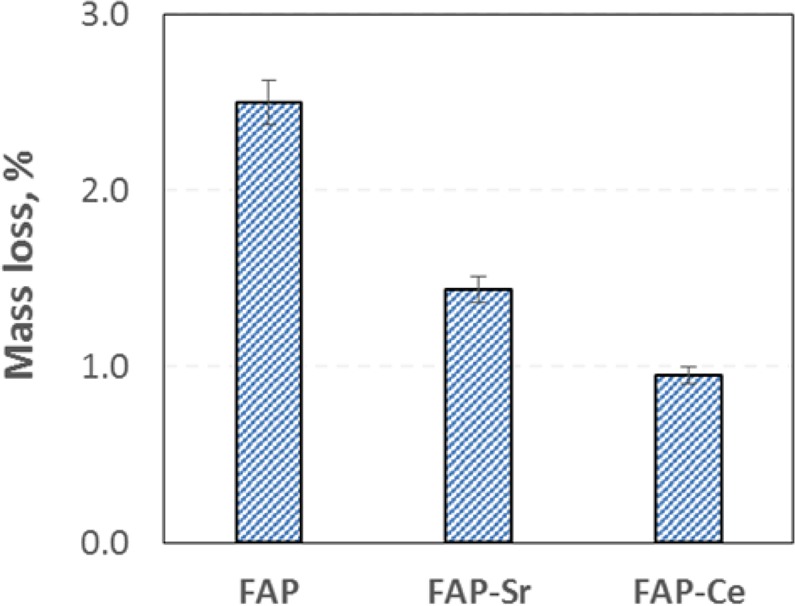
Mass loss of minerals after 48 h in a phosphate buffer (3 pellets of each mineral were tested, with initial mass of 0.25 g/pellet).

For understanding the antibacterial activity of each mineral, it is essential to know the corresponding dissolution rate in physiological conditions (pH = 7.4 and temperature of 37 °C). In Fig. 5, the weight % mass loss of each mineral (in powder form) in phosphate buffer (pH = 7.4) after 48 h is presented. In general, the resorption rate for all the materials is quite low which may be beneficial for bone regeneration since, the healing of the hard tissue often takes longer than 6 weeks. The higher mass loss was found for the undoped fluorapatite (2.5%) and it was followed by the Sr^2+^ doped material (1.4%). The lowest dissolution rate was observed for FAP-Ce (0.97%).

After testing different concentrations of the powders with four different bacteria, we were able to identify the half maximal inhibitory concentration (IC_50_) of each material (Table 4). It was found that the undoped fluorapatite has the higher inhibition potential for all the bacteria tested since it presents the lowest IC_50_ values (around 110 μg/ml for *E*. *coli*, 112 μg/ml *for S*. *aureous* and 119 μg/ml for *B*. *subtilis* and 156 μg/ml for *B*. *cereus*). Doping fluorapatite with Ce^3+^ has no particular effect on the antibacterial properties since, the IC_50_ values of FAP-Ce are very close to that of FAP, both for the Gram-negative (*E*. *coli*) and the Gram-positive (*S*. *aureus*, *B*. *cereus* and *B*. *subtilis*) bacteria. On the other hand, doping with Sr^2+^, seems to reduces the antibacterial potential of the mineral since there is a clear increase of the IC_50_ for all the bacteria tested. As it is presented in Table 4, the IC_50_ values for the FAP-Sr powder found to be between 30 and 60% higher than these of the undoped fluorapatite (FAP).

**Table 4:** Half maximal inhibitory concentration (IC_50_) value of FAP, FAP-Sr and FAP-Ce for *E*. *coli*, *S*. *aureus*, *B*. *subtilis and B*. *cereus*.

*IC*_*50*_ (*μg*/*ml*)	*E*. *coli*	*S*. *aureus*	*B*. *subtilis*	*B*. *cereus*
FAP	110 ± 7.5	112 ± 6.2	119 ± 7.3	156 ± 10.1
FAP-Sr	141 ± 6.0	149 ± 10.1	191 ± 11.2	239 ± 13.1
FAP-Ce	107 ± 7.1	135 ± 9.3	133 ± 8.1	165 ± 11.5

**Figure 6:**
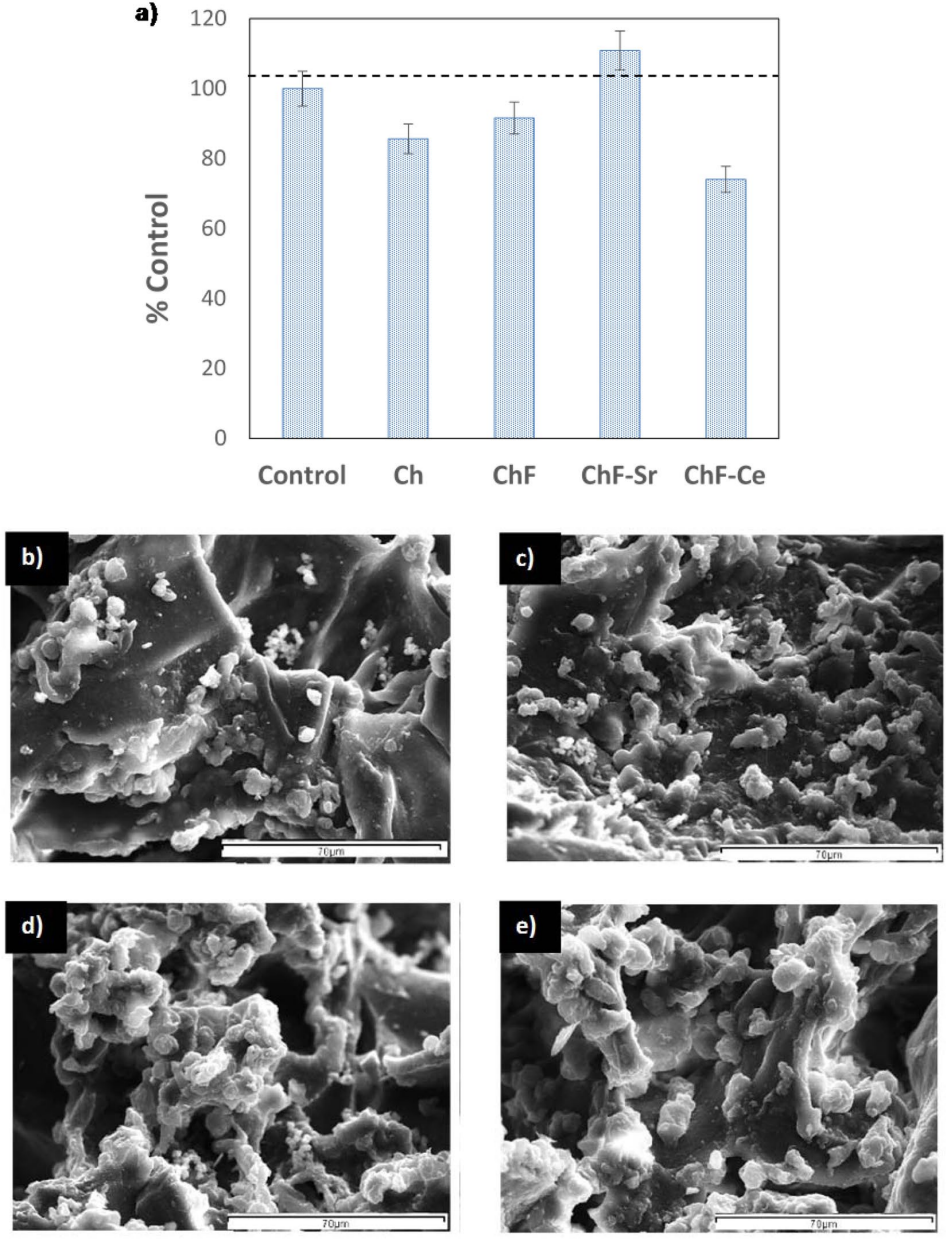
Interaction of the fabricated scaffolds with DPSCs. (**a**) Measurements of metabolic activity (MTT assay); SEM micrographs of the DPSCs on (**b**) CH, (**c**) CHFAP, (**d**) CHFAP-Sr) and (**e**) CHFAP-Ce scaffolds. Cells migrate from the culture media and occupy the pores of the scaffold (scale bars of 70 μm).

Measurements of metabolic activity (MTT) were carried out for all the chitosan scaffolds in order to determine the toxicity of our materials. Figure 6a shows statistically significant reduction in cell viability when compared with the control only in the presence of the FAP-Ce. However, this reduction is not so high enough to deem the material toxic. In general, these results indicate good cytocompatibility of the synthesised minerals. This is also supported by the SEM images in Fig. 6b–e where the successful attachment of the cells was observed following the completion of the cultures. The morphology of the cells on the scaffolds confirms the results of MTT for their non-cytotoxic effect of the tested materials on the biological functions of the DPSCs.

**Figure 7:**
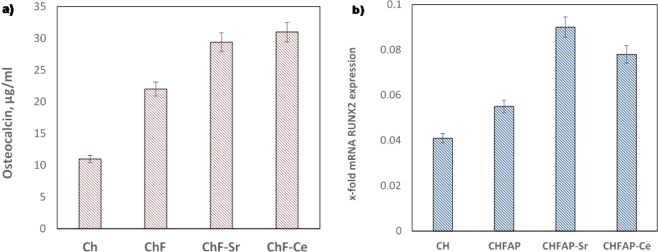
Differentiation of the DPSCs to osteoblasts. (**a**) Concentration of osteocalcin on each scaffold. (**b**) Expression of RUNX2 gene.

To determine the potential differentiation of the stem cells to osteoblasts, the concentration of osteocalcin after 4 days was measured. As it is depicted in Fig. 7a, the lowest concentration was measured for the chitosan scaffolds with no containing minerals (11 μg/ml). For the fluorapatite (FAP) containing scaffolds, the concentration of osteocalcin was considerably higher (22 μg/ml) but the maximum value was found for the scaffolds with the Ce doped mineral (31 μg/ml). These are in accordance with the findings from RUNX2 gene expression, where the highest values measured for Sr^2+^ and Ce^3+^ containing scaffolds (Fig. 7b). Based on these results is clear that adding Sr^2+^ or Ce^3+^ favours the differentiation of the pulp stem cells to osteoblasts, which indicates that the bone regenerative potential of the synthesized fluorapatite is enhanced (as a result of doping with these two types of ions).

## Discussion

In this work we demonstrated the incorporation of Sr^2+^ and Ce^3+^ ions into the fluorapatite lattice, as a result of the cationic substitution of Ca^2+^ ions. The X-ray powder diffraction analysis and FTIR spectra imply that there are not major changes in the crystalline lattices of the doped minerals when compared with the undoped fluorapatite, and this is justified if we consider the similar ionic radii of Ca^2+^ with Sr^2+^ and Ce^3+^. However, this may change if the doping concentration is increased (in this work 5% mol). For example, in^15^ it was found that as the amount of Ce^3+^ increases, positions of Bragg peaks in hydroxyapatite changed and became broader, indicating that the resulting minerals were less crystalline than the undoped material.

The minerals synthesized herein were found to have significant antibacterial activity, and particularly the undoped and the Ce-doped fluorapatite (FAP and FAP-Ce) which present similar behaviour. In a systematic work of Wu *et al*.^27^, the IC_50_ of hydroxyapatite NPs for *E*. *coli* and *S*. *aureus* was found to be 29 mg/ml and >100 mg/ml respectively. For the fluorapatites presented in this work the IC_50_ values for the same bacteria are almost one order of magnitude lower (Table 4) making apparent the high antibacterial potential. Although there are numerous reports about the general antibacterial effect of fluoride, little work has been published regarding the antibacterial action of fluorapatite (fluoride incorporated in calcium phosphate). In a recent work of Alhilou *et al*.^28^, fluorapatite was investigated as a potential coating material for dental scaffolds/implants and it was demonstrated that it significantly reduces the viability of the adherent bacteria for all the bacterial strains tested (i.e. *P*. *gingivalis*, *F*. *nucleatum*, *A*. *actinomycetemcomitans*). These results are in agreement with the findings of the present work where, we demonstrated that the FAP minerals have the potential to inhibit bacterial growth both for Gram-negative and Gram-positive bacterial strains (Table 4). Doping FAP lattice with Sr^2+^ resulted in the attenuation of the inhibition effect almost for all the cases tested (higher IC_50_ as is presented in Table 4). As already discussed in introduction, the antibacterial potential of Sr^2+^ is disputable since there are reports supporting both cases (e.g.^12,14^), and thus the exact mechanism of how Sr^2+^ ions affect different bacteria types remains unclear. One important factor for explaining the different behaviour of FAP and FAP-Sr is the lower degradation rate of the latter. Since the mass loss of the Sr^2+^-doped mineral is lower than that of FAP, less F ions (which are responsible for the antibacterial effect of fluorapatite) are released, allowing higher bacteria growth rates. Although doping with Ce^3+^ results even lower degradation rate, the antibacterial effect of the FAP-Ce mineral found to be almost the same with that of FAP. From the literature it is evident that doping with Ce^3+^, significantly improves the antibacterial ability of the apatite minerals^15,29^. Consequently, we may assume that in the case of FAP-Ce, the observed inhibition is the result of the synergistic action of the F^−^ and Ce^3+^ ions.

To check the regenerative potential, the mineral powders used as fillers in chitosan scaffolds and tested with human dental pulp stem cells (DPSCs). Based on the analysed concentrations of osteocalcin and the RUNX2 expression, the scaffolds containing the FAP-Sr (29.4 μg/ml osteocalcin) and the FAP-Ce (31.0 μg/ml osteocalcin) induced considerably higher osteogenic differentiation of the pulp stem cells than the undoped fluorapatite (21.0 μg/ml osteocalcin) or the chitosan scaffolds without mineral fillers (11.1 μg/ml osteocalcin). The ability of Sr^2+^ ions to increase the bioactivity of apatite biomaterials and to promote the osteogenic gene expression is well documented in literature. In agreement with the results of other reports^30^, we demonstrated that the presence of Sr^2+^ in the scaffold enhances the osteogenic proliferation of human dental pulp stem cells (DPSCs), and thus it can be an effective option for the regeneration of dental tissues (e.g. coating of dental implants). The addition of cerium Ce^3+^ found to have a similar effect. In general, due to the similar ionic radii with Ca^2+^, cerium can present diverse biological effects^31^ and it is well known for its pharmaceutical applications (e.g. antiemetic and antiseptic). However, as it is concluded from our results it can also promote the osteogenic differentiation of DPSCs when is incorporated into fluorapatite lattice. Although there are not many reports about the interaction of Ce with DPSCs, Ce^3+^ found to enhance the osteogenic proliferation of bone mesenchymal stem cells BMSCs when was added to hydroxyapatite coatings^32^.

## Conclusions

In this work we investigated the incorporation of Sr^2+^ and Ce^3+^ ions into fluorapatite powders aiming to a material with enhanced antibacterial properties and regenerative potential. The most important findings of this work can be summarised in the following:

- In terms of materials synthesis, we demonstrated that fluorapatite can be doped with low concentrations of Sr^2+^ and Ce^3+^ without affecting its crystal structure.
- The undoped fluorapatite found to have better antibacterial response both for Gram- positive and Gram-negative bacteria. The antibacterial activity is reduced with the addition of Sr^2+^ but it remains almost the same when doping with Ce^3+^.
- The osteogenic potential of the Ce^3+^ doped mineral found to be the same with that of the Sr^2+^ doped mineral. In particular, after experiments with DPSCs the same concentration of osteocalcin was measured for both materials, indicating similar degree of osteogenic differentiation.

Based on previous works in literature we expected the good antibacterial properties of fluorapatite and the exceptional osteogenic potential of Sr^2+^ doped minerals. In this work for the first time we demonstrated that the incorporation of Ce^3+^ into fluorapatite lattice, results a material which combines both of the aforementioned properties and results into an improved biomaterial. Although more systematic work is needed (e.g. optimising the doping concentration), cerium doped fluorapatite seems to be a promising biomaterial that could provide solutions for significant clinical conditions where bacterial contamination is a critical problem as for example peri-implantitis.
